# Graph MADDPG with RNN for multiagent cooperative environment

**DOI:** 10.3389/fnbot.2023.1185169

**Published:** 2023-06-29

**Authors:** Xiaolong Wei, WenPeng Cui, Xianglin Huang, LiFang Yang, Zhulin Tao, Bing Wang

**Affiliations:** ^1^Department of Energy Efficiency, Beijing SmartChip Microelectronics Technology Co., Ltd., Beijing, China; ^2^State Key Laboratory of Media Convergence and Communication, Communication University of China, Beijing, China

**Keywords:** multiagent, MADDPG, RNN, graph convolutional network, attention

## Abstract

Multiagent systems face numerous challenges due to environmental uncertainty, with scalability being a critical issue. To address this, we propose a novel multi-agent cooperative model based on a graph attention network. Our approach considers the relationship between agents and continuous action spaces, utilizing graph convolution and recurrent neural networks to define these relationships. Graph convolution is used to define the relationship between agents, while recurrent neural networks define continuous action spaces. We optimize and model the multiagent system by encoding the interaction weights among agents using the graph neural network and the weights between continuous action spaces using the recurrent neural network. We evaluate the performance of our proposed model by conducting experimental simulations using a 3D wargame engine that involves several unmanned air vehicles (UAVs) acting as attackers and radar stations acting as defenders, where both sides have the ability to detect each other. The results demonstrate that our proposed model outperforms the current state-of-the-art methods in terms of scalability, robustness, and learning efficiency.

## 1. Introduction

The traditional reinforcement learning technique works well in solving problems with a small sample space. However, its efficacy is poor when it comes to addressing the challenge of expanding the state space and action space. Following Silver's implementation of AlphaGo (Silver et al., 2016) to defeat the international chess champion, deep reinforcement learning has gradually entered academic and industrial fields and has outstandingly achieved milestones in various areas. Though numerous problems need to overcome for multi-agent learning, deep reinforcement learning presents itself as an excellent solution. As far back as 2000, Stone and Veloso (2000) analyzed multi-agent systems through the machine learning prism and mainly focused on four types of agents–whether agents are homogeneous or heterogeneous, or they are communicative. On the other hand, early review articles (Gordon, 2007) analyzed multi-agent learning from different standpoints, leading to the identification of four major issues of multi-agent learning, including problem description, distributed artificial intelligence, game equilibrium, and agent modeling (Shoham et al., 2003). Tošić and Vilalta (2010) proposed a framework for multi-agent reinforcement learning and meta-learning. Similarly, Tuyls and Stone (2017) analyzed online reinforcement learning for individual utility, social welfare, and co-evolution. Tuyls and Weiss (2012) emphasized that the integration of swarm intelligence, co-evolution, transfer learning, and non-stationarity techniques should be considered when developing multi-agent learning methods.

Deep reinforcement learning is the combination of reinforcement learning and deep learning, and multiagent reinforcement learning is clustered with multiple agents. The value-based deep reinforcement learning algorithm can fit the continuous state space, but the value-based method cannot solve the problem of continuous action space. Therefore, to solve this problem, many researchers have proposed a variety of policy gradients based on the actor-critic (Konda, 2005) framework. Policy gradient includes stochastic policy gradient methods and deterministic policy gradient algorithms. The traditional policy gradient algorithm is an on-policy method that cannot overcome the problem of low sample utilization. A3C (Mnih et al., 2016), TRPO (Schulman et al., 2015), and PPO (Schulman et al., 2017) have greatly simplified the difficulty of understanding and have ensured the effect of the algorithm. The deterministic policy gradient (Silver et al., 2014) can directly obtain the gradient of the policy through chain rule derivation. Then, the deterministic policy gradient algorithm was combined with deep learning, and a deep deterministic policy gradient (DDPG) was proposed (Lillicrap et al., 2016). Heess et al. (2015) combined DDPG and long short-term memory to propose RDPG, which optimized the reinforcement learning process and the efficiency of consecutive sequences. Due to the limitation of the Nash equilibrium, DDPG does not perform well in the multiagent-enhanced environment. Compared with the single-agent environment, the multiagent environment is unstable. There are many uncertainties in the training process. According to the literature on robots, which includes the studies mentioned in references (Lowe et al., 2017; Chen et al., 2021, 2022; Wang et al., 2021), traditional control and deep reinforcement algorithms have been widely applied to lots of areas, while recent research has increasingly focused on the application of reinforcement learning techniques. MADDPG (Lowe et al., 2017) is an extended method from DDPG that adds centralized training distributed execution. Based on MADDPG, many studies have made great contributions in recent years. Wang et al. (2020) proposed RMADDPG by combining long short-term memory and MADDPG. Iqbal and Sha (2019) proposed a method by combining attention and MADDPG. Significant approaches (Jiang and Lu, 2018; Yathartha and Enna Sachdeva, 2019; Wei et al., 2022) have made great improvements in MADDPG. The MADDPG (Mordatch and Abbeel, 2018) environment is based on time series. The recurrent neural network (RNN; Zaremba et al., 2014) is calculated from left to right or from right to left. This mechanism is accompanied by the model mentioned (Vaswani et al., 2017) parallel ability and long-term dependence. In the context of deep reinforcement learning, the number of agents in the environment is subject to change. The dimensions of the agent's observation state will change, the input of the neural network will change, and the neural network cannot be reused. The change in the number of agents of the environment will lead to a change in the optimal policy. Therefore, most reinforcement learning algorithms cannot be effectively expanded in multiagent systems, their application range is extremely limited, and they can only be applied to a fixed number of multiagent tasks.

Graph convolutional network (Gama et al., 2018; Zhou et al., 2020) has appeared in recent years. Based on the assumptions of high dimensional and partial information, Arbaaz constructed a proposed graph policy gradient (GPG; Khan et al., 2019). GPG solves the learning problem of controlling multiple agents and effectively transfers the policy when the number of agents changes. For the purpose of directly combining a graph neural network (GNN) and label propagation algorithm (LPA), Shi et al. (2020) proposed a graph transformer network. Transformers (Vaswani et al., 2017) are widely used in many fields (Devlin et al., 2018; Vaswani et al., 2018; Carion et al., 2020). Adopting multihead attention (Bahdanau et al., 2014) into graph learning could take into account the case of edge features. The multiagent reinforcement learning method MADDPG does not consider the relationship between multiple agents and continuous actions on the timeline.

This study proposes a novel solution to learning individual control policies for multiple agents by leveraging their underlying graph structure. In environments with N agents, each agent only receives partial observations of the environment and must interact with a subset of other agents to effectively learn a control policy. Identifying the appropriate subset of neighbors is a challenging problem, particularly as the number of agents increases, and it is necessary to ensure that the cardinality of the subset remains fixed or grows slowly to maintain scalability.

To address these challenges, we draw inspiration from GPG's approach of sequentially composed layers with linear filters to extract local features and reduce dimensionality. However, GPG has limitations as it is only suitable for symmetry problems and not adaptable to more complex, changeable environments. To overcome these limitations, we introduce graph convolutional MADDPG with attention, which uses sequentially composed layers that regularize the linear transform in each layer to become a graph convolution with a bank of graph filters, with the filter weights learned by minimizing a cost function.

Our approach exploits the underlying graph structure among agents, facilitating effective learning of control policies with scalable and efficient computation by leveraging attention mechanisms to focus on relevant information for each agent and using graph convolution to capture the graph structure among the agents. Building on the success of RMADDPG, we propose a novel multiagent collaboration model based on a graph convolutional network with an attention mechanism. The graph convolutional network defines the relationship function between agents and can handle high-dimensional problems. Furthermore, by integrating recurrent neural networks (RNNs) into our approach, we can model continuous timeline actions, capture temporal dependencies between actions, and ensure that the agents' policies remain consistent over time. Overall, our proposed approach considerably advances the development of scalable and efficient algorithms for learning individual control policies for multiple agents. By utilizing attention mechanisms, graph convolution, and RNNs, we effectively learn individual control policies for multiple agents by exploiting their underlying graph structure.

This study presents three major contributions. First, we have developed a 3D environment for electronic warfare simulations. Second, we introduce a novel method based on a graph convolutional network with an attention mechanism to effectively define the relationship function between agents. Third, we propose a new method based on recurrent neural networks to model continuous actions. The related works, such as POMDPs, MADDPG, recurrent MADDPG, and transformer, are discussed in Section 2. Section 3 provides a detailed description of our proposed method. The experimental and computational results are presented in Section 4, which confirm the effectiveness of our approach. Finally, Section 5 summarizes our main findings and suggests directions for future research.

## 2. Background

### 2.1. POMDP

In reinforcement learning enrichment, decisions are made according to the current actual state of the system, but in many cases, the exact state of the system is difficult to obtain. The partially observable Markov decision process (POMDP) is used to describe the hidden system states and uncertain behavior effects. The POMDP is a seven-tuple, *S, A, T*, Ω, *R, O*, and γ, where

1. *S* represents the state space and contains all the information we need in the environment.
2. *A* represents the action space of the agent, including all potential actions.
3. *T* represents the state transfer function of the environment.
4. Ω represents the observation function and maps the environment state to the observation space of the agent.
5. *R* represents the reward function and evaluates the actions of the agent.
6. *O* represents the observation space and is the observable space for an agent.
7. γ is the discount factor.

When you take in information and act on the POMDP, the current state changes accordingly. By anticipating the state and reacting to your environment, an agent can better determine its own state by the function $\tau =\left\{o_{0},a_{0},r_{1},o_{1},a_{1},r_{2}...\right\}\in T$. The agent chooses appropriate actions according to the states, and the actions taken by the agent will affect the environment. Thus, they form a closed loop and constantly influence each other.

The deep deterministic policy gradient (DDPG; Lillicrap et al., 2016) is a type of actor-critic (Konda, 2005) method. For DDPG, the idea of DQN (Mnih et al., 2013) has not changed, but the application has changed. Compared with DQN, DDPG mainly solves the problem of continuous action prediction. It can be seen that the implementation difference between continuous and discrete actions is only in the choice of the final activation function. Therefore, DDPG has made some improvements on the algorithm inherited from DQN. To extend DDPG to multiagent systems, the MADDPG (Lowe et al., 2017) based on DDPG is proposed. The MADDPG algorithm is more efficient than DDPG for multiagent systems. The optimal policy obtained through learning can give the optimal action only by using local information during application. There is no need to know the dynamic model of the environment and special communication requirements. The MADDPG can be used not only in cooperative environments but also in competitive environments.

MADDPG, while a powerful deep reinforcement learning framework for multi-agent scenarios, is not without its drawbacks. Specifically, its computing mode and communication mechanisms are limited, hampering its ability to perform optimally in some cases. However, these challenges also create significant opportunities for growth and enhancement in the field of deep reinforcement learning, as researchers work to overcome these obstacles and improve MADDPG's overall performance. Angeliki (Lazaridou et al., 2016) proposed a framework for language learning that relies on multiagent communication. In MADDPG, multiagents can only share their actions and observations in the training stage through the critic, and there is no communication in the execution stage, so the MADDPG algorithm cannot be well qualified for some tasks that require communication. MD-MADDPG (Pesce and Montana, 2020) was proposed as a framework for multiagent training using DDPG, and MD-MADDPG can learn the explicit communication protocol through a memory device. Based on Dropout (Srivastava et al., 2014), to reduce the dimensions, the Message-Dropout MADDPG (Kim et al., 2019) was proposed. For sharing the globe parameter, BiCNet (Peng et al., 2017) was proposed with a bidirectional recursive neural network. Iqbal and Sha (2019), Liu Y. et al. (2020), and ATT-MADDPG (Mao et al., 2018) improved the MADDPG performance by an attention mechanism.

### 2.2. Graph convolutional networks

Inspired by graph policy gradient (GPG; Khan et al., 2019), we use the convolution operation proposed by Gama et al. (2020) in GPG (Khan et al., 2019), which is different from the traditional convolutional neural network (CNN) in that the signal *x* is transferred to the spectral domain, and then, the data of multiple neighborhoods are added. Among them, **S** is the Laplacian matrix of the graph, and *h*_*k*_ is the filter weight. In the process of creating the network, the convolution operation in the fully connected layer is first replaced by the graph convolution operation. The linear transformation outputs the result of the next layer. Graph convolutional network applies deep learning to the structure of Euclidean space ℝ^*n*^ to construct the relationship between vertices and edges representing objects 𝔾 = (*V, E*), where *V* is a node set and *E* is an edge set, which shows good robustness and interpretability. Therefore, it is an effective way to model the interaction mode between multiple agents through graph topology. Here, we define each agent as a node. If the distance between the agents is less than ϵ, there is an edge between the two agents. This graph is used as a data vector $X={\left[x_{1},...x_{N}\right]}^{T}$ ratio, where *x*_*n*_ is the state representation of agent n. The vector *X* is used as the input of the graph convolutional network. After multiple convolutional layers, the graph convolutional network can learn the features between nodes. The output of the graph convolutional network is π = [π_1_, ..., π_*N*_], which is the policy of the ith agent. Finally, reinforcement learning uses the policy gradient (Sutton et al., 2000) to update the weight of the policy network.

The problem stated by the above theorem is that as long as the topology of the graph remains unchanged, the output of the graph convolution will not change under the reordering of the graph nodes. Intuitively, in the graph, several nodes with the same graph neighborhood can share the same set of filters. When learning the control of a large number of agents, this feature helps reduce the conditionality of the problem.

**Algorithm 1:** Graph deep deterministic policy gradient based on RNN for *N* agents.

**Input:** Input tensor $x_{i}^{t}$, *t* ∈ [0, *M*], *i* ∈ [0, *N*], M is the length of episodes, N is the number of agents, and *S*_*t*_ is adjacency matrix.	**Output:** $a_{i}^{t}$ is expect action.
1: for episode = 1 to M **do**
2: Initialize a random process N for action exploration
3: Receive initial state *s* initialize empty history *h*
4: for t = 1 to max-episode-length **do**
5: for each agent i select action:
6: obtain input tensor $x_{i}^{t}$ and adjacency matrix *S*_*t*_
7: $a_{i}=\mu_{\theta_{i}}\left(o_{i},h_{i}\right)+N_{i}$
8: Execute actions *a* = (*a*_*i*_, ..., *a*_*N*_)
9: Get reward *r*, new state *s*′, new history *h*′
10: store (*s, h, a, r, s*′, *h*′) in replay buffer D
11: for agent *i* = 1 to N **do**
12: Sample a random mini-batch of S samples
13: for t = 1 to hierarchical-layers **do**
14: *x*′ = *Attention*(*L*(*H*(*x*′)))
15: end **for**
16: $Y^{i}=r_{i}^{j}+\gamma Q_{i}^{\mu^{\prime }}\left(x^{\prime }\right){|}_{a_{k}^{\prime }=\mu_{k}^{\prime }\left(s_{k}^{j},h_{k}^{j}\right)}$
17: Update critic by minimizing the loss:
18: $L\left(\theta_{i}\right)=\frac{i}{S}{\left(\sum_{j}y^{i}-Q_{i}^{\mu }\left(x\right)\right)}^{2}$
19: Update actor:
20: $\nabla_{\theta_{i}}J\approx \frac{i}{S}\underset{j}{\sum }\nabla_{\theta_{i}}\mu_{i}\left(s_{i}^{j},h_{i}^{j}\right)\nabla_{a_{i}}Q_{i}^{\mu }\left(x\right){|}_{a_{k}=\mu_{k}\left(s_{k}^{j},h_{k}^{j}\right)}$
21: end **for**
22: Update target network parameters
23: for each agent *i*
24: $\theta_{i}^{\prime }\leftarrow \tau \theta_{i}+\left(1-\tau \right)\theta_{i}^{\prime }$
25: end **for**
26: end **for**

## 3. Methods

### 3.1. Multihead attention

Attention (Bahdanau et al., 2014) was proposed by Bengio in 2014 and has been widely used in various fields of deep learning in recent years. For example, it was used in the field of computer vision to capture the receptive field on the image or to locate key tokens or features in NLP. The BERT (Devlin et al., 2018) method for generating word vectors recently proposed by the Google team has achieved significant improvements in NLP tasks. The most important part of the BERT algorithm is a milestone for the transformer concept. The transformer with self-attention as the basic unit proposed by Vaswani enables attention to be successfully applied. Traditional CNNs and RNNs are abandoned in transformers, and the entire network structure is entirely composed of an attention mechanism. More precisely, a transformer consists of self-attention and a feedforward neural network. In the studies mentioned in references (Iqbal and Sha, 2019; Wei et al., 2021), many contributions to MADDPG and the attention mechanism were made, which achieved remarkable results.

To better learn the relationship between agents in the graph convolutional network, inspired by graph transformers (Shi et al., 2020), we introduced attention to graph convolutional networks. Transformers have been proven to have unprecedented value in natural language processing (NLP) and image signal processing. The graph attention network algorithm (Veličković et al., 2018) was proposed by combining an attention machine and a graph convolutional network. Graph attention network solves the problems of graph convolutional network and has achieved state-of-the-art effects on many tasks. Shi et al. (2020) proposed the graph transformer algorithm for the first time and applied it to the semisupervised classification task to achieve state-of-the-art effects. Based on previous studies, we applied its multihead attention to graph learning and combined the transformer and graph policy gradient (GPG) algorithm. Here, we define the given node as $H^{\left(l\right)}=\left\{h_{1}^{\left(l\right)},h_{2}^{\left(l\right)},...,h_{n}^{\left(l\right)}\right\}$. We define the multihead attention for each edge from *j* to *i* as follows:


(1) $$\begin{array}{l} \begin{array}{c} q_{c,i}^{\left(l\right)}=W_{c,q}^{\left(l\right)}h_{i}^{\left(l\right)}+b_{c,q}^{\left(l\right)}\\ k_{c,j}^{\left(l\right)}=W_{c,k}^{\left(l\right)}h_{j}^{\left(l\right)}+b_{c,k}^{\left(l\right)}\\ e_{c,ij}=W_{c,e}e_{ij}+b_{c,e} \end{array} \end{array}$$

where $q_{c,i}^{\left(l\right)},k_{c,j}^{\left(l\right)},e_{c,ij}\in {\mathbb{R}}^{d_{s}\times d_{s}}$ are the weight metrics, and the attention is computed as follows:


(2) $$\begin{array}{l} a_{c,ij}^{\left(l\right)}=\frac{\langle q_{c,i}^{\left(l\right)},k_{c,j}^{\left(l\right)}+e_{c,ij}\rangle }{\sum_{u\in N_{\left(i\right)}}\langle q_{c,i}^{\left(l\right)},k_{c,u}^{\left(l\right)}+e_{c,iu}\rangle } \end{array}$$

Multihead attention is applied to multiagent reinforcement learning: $\left\{D^{k}\right\}$. The calculation is a combination of hidden state $h_{n}^{\left(l\right)}$:


(3) $$\begin{array}{l} \text{MultiHead}=\text{Concat }\left(h_{1}^{\left(l\right)},h_{2}^{\left(l\right)},...,h_{n}^{\left(l\right)}\right)W^{O} \end{array}$$

For the query, the key and edge matrices are $q_{c,i}^{\left(l\right)},k_{c,j}^{\left(l\right)},e_{c,ij}\in {\mathbb{R}}^{M\times d_{s}}$. The weight matrix is expressed as $W^{O}\in {\mathbb{R}}^{d_{s}h\times d_{s}}$. The graph transformer is proposed for the semisupervised classification task to achieve state-of-the-art effects. Similar to the graph attention network, the graph transformer adds attention after the output layer, calculates the average value of the multihead, and removes the non-linear transformation.

### 3.2. RNN position embedding

The transformer embodies each word but does not contain relative position information. Recurrent neural network (RNN) processes the sentences in order. In the transformer, all the words of the input sentence are processed at the same time, without considering the ordering and position information of the words. In this regard, the author of the transformer proposed a method of adding “positional encoding” to solve this problem. Positional encoding allows transformers to measure information related to word position. As shown in Equation (4), *e*_*p*_ is the positional encoding for the transformer, where *p* refers to the position of the word in this sentence, and *i* refers to the embedding dimension.


(4) $$\begin{array}{l} \begin{array}{l} \begin{array}{c} \;e_{p}[i]=sin(p/10000^{2i/d})\\ e_{p}[2i+1]=cos(p/10000^{2i/d}) \end{array} \end{array} \end{array}$$

The self-attention model represented by the transformer has positional permutation invariance. Disrupting the word model in the sentence will obtain the same characteristics. For this reason, this type of model needs to add “location coding” so that the model can recognize what words are in any location. For NLP, we can use Equation (4) to encode the position of the word, but in the reinforcement learning environment, the spatial dimension will have much uncertainty compared with the word. For any language, the number of words will be approximate. Although it can theoretically handle long sentences, because it is designed by humans rather than automatically learning from data, it may not be effective. In the reinforcement learning environment, the state and action of each step are special and independent, they are also uncertain, and the state space is infinite. To this end, we need to find a learnable model. Liu X. et al. (2020) proposed FLOATER which uses neural ordinary differential equations (ODEs) to model positional coding. The position coding based on the recursive model also has better extrapolation. At the same time, it has better flexibility than the position coding of the trigonometric function. Liu proved that the position coding of the trigonometric function is a certain FLOATER, i.e., a special solution. Here, we define {_*p*_*i*_}*i* ∈ {1, 2, ...*N*}_ as a position in each episode. where the *h*_*i*_ is the hidden state, and *p*_*i*_ is the hierarchical connection between adjacency layers.


(5) $$\begin{array}{l} p_{i+1}=RNN\left(h_{i},p_{i}\right) \end{array}$$

In the reinforcement learning environment, each time sequence corresponds to an RNN memory block. RNN accumulates context information step-by-step in each episode. The low level contains *N*−*th* RNN encoders, and *N*−*th* RNNs correspond to the state of *N*−*th* time points with the current time as the end point. Based on the RNN to obtain *i*−*th*, the corresponding hidden state *h*_*i*_ can be obtained, and then, the step-level attention mechanism is adopted to obtain the sequence representation *r*_*i*_ through the weighted sum of the hidden states of all steps in the sequence.

### 3.3. Graph MADDPG with RNN and attention

**Figure 1:**
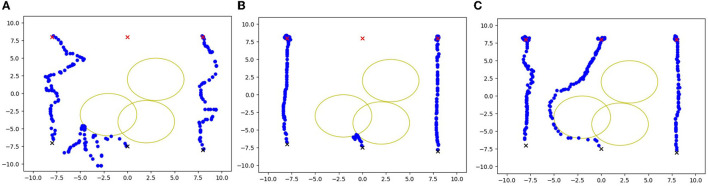
The motion trajectory of the agent, where **(A)** is the MADDPG, **(B)** is the GPG, and **(C)** is the method proposed.

As show in the Figure 1, we propose a framework for graph Multi-Agent Deep Deterministic Policy Gradient (MADDPG) with transformers. In this framework, graph convolutional networks are utilized to aggregate information between nodes and their y-hop neighbors. We then combine the current state and the previous state's output as inputs for graph convolutional network and transformer embedding layers.

**Figure 2:**
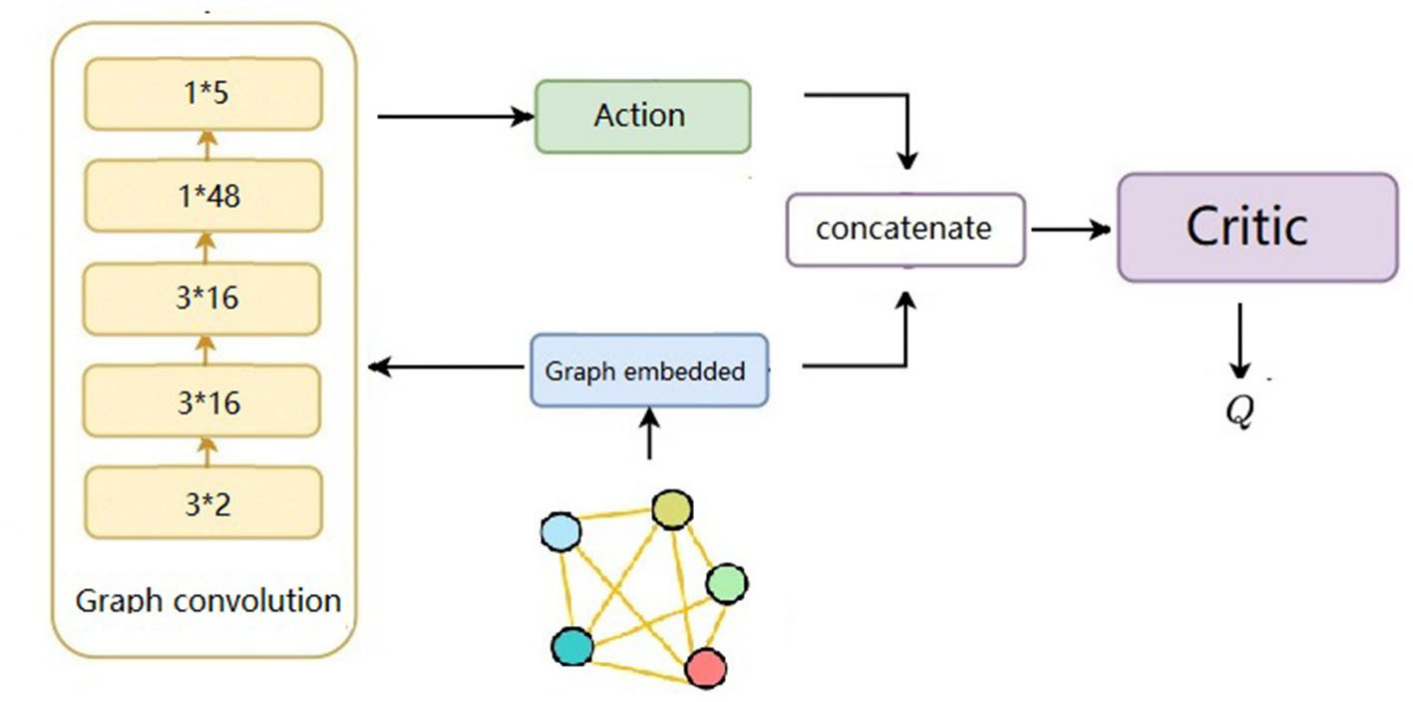
The input of the neural network is *n* × (*x, y*), where the *n* equals to the number of agents, the output can be obtained after two graph convolutional network layers and two fully connected layers in sequence.

Learning the critic and actor for each agent by selectively paying attention to information from other agents underlies much of this study. Figure 2 illustrates the main components of our approach. The proposed method uses 𝔾 = (*V, E*) to compute the graph and the embedding vectors *p*_*i*_ of each agent to compute the RNNs. We define the *A* as a series of actions *a*_1_, *a*_2_, ...*a*_*N*_. The Q-value function *Q*_*i*_(**x**, *p*_*i*_, *a*_*i*_) and the action *a*_*i*_ = **μ**_*i*_(*o*_*i*_, *p*_*i*_) are determined based on this information. During the process, all critics are updated simultaneously to minimize the joint regression loss function. The critic network generates input and output as defined below. The proposed method is an effective tool for improving accuracy and efficiency while enhancing the overall performance.


(6) $$\begin{array}{l} L\left(\theta_{i}\right)={\text{𝔼}}_{\text{x},a,r,{\text{x}}^{\prime }}\left[{\left(Q_{i}^{\mu }\left(\text{x},p_{i},a_{1},\ldots ,a_{N}\right)-y\right)}^{2}\right] \end{array}$$

where


(7) $$\begin{array}{l} y=r_{i}+\gamma Q_{i}^{\mu^{\prime }}\left({\text{x}}^{\prime },p_{i}^{\prime },a_{1}^{\prime },\ldots ,a_{N}^{\prime }\right){|}_{a_{j}^{\prime }=\mu_{j}^{\prime }\left(o_{j},p_{i}\right)} \end{array}$$

Where *y*^*i*^ is the *Q* generated by the intelligent agent choosing the path planning behavior according to the policy and μ under observation *o*. Algorithm training is carried out with the goal of maximizing the reward value while minimizing the *L*(θ_*i*_) function. The individual policies are updated by ascent with the following gradient:


(8) $$\begin{array}{l} D=\nabla_{\theta_{i}}\mu_{i}\left(a_{i}|o_{i},p_{i}\right)\nabla_{a_{i}}Q_{i}^{\mu }\left(\text{x},p_{i},a_{1}\ldots a_{N}\right) \end{array}$$

(9) $$\begin{array}{l} \begin{array}{c} \nabla_{\theta_{i}}J\left(\mu_{i}\right)={\text{𝔼}}_{\text{x},a\sim D}\left[D{|}_{a_{i}=\mu_{i}\left(o_{i},p_{i}\right)}\right] \end{array} \end{array}$$

Here, $Q_{i}^{\mu }\left(\text{x},p_{i},a_{1}\ldots a_{N}\right)$ is a centralized action-value function that takes as input the actions of all agents, μ_*i*_ is the set of policies with delayed parameters, *a*_1_…*a*_*N*_, in addition to some state information x, and outputs the *Q*-value for agent i. In the simplest case, *x* could consist of the observations of all agents, *x* = (*o*_1_, …, *o*_*N*_); however we could also include additional state information if available. Agents can have arbitrary reward structures, including conflicting rewards in a competitive setting. Unlike in the MADDPG, the other agents' observations are sampled from the linear data. The method that we propose magnifies information likely to contain relationships between agents, making the use of RNNs and graphs with transformers learned from correlations between agents and time-associated sequences.

In reinforcement learning, the complexity of the environment often leads to an increase in both the state space and action space. To deal with high-dimensional, continuous, or partially observable state spaces, specific reinforcement learning algorithms, and techniques, such as value function approximation and policy gradient, are necessary. The complexity of the action space also affects the algorithm's feasibility and efficiency. Therefore, selecting appropriate algorithms and techniques is crucial to handle high-dimensional state and action spaces in complex reinforcement learning problems, improving the algorithm's feasibility and efficiency. As for the details of the model, the state space and action space are not explicitly described in the provided information. However, the model's architecture includes two hidden layers of graph convolution with a channel size of 16 each and an input layer with three nodes, the channel size of action is 5. The loss function used is MSEloss, with an update factor of 0.001 and a learning rate of 0.01. The batch size is set to 1,024, and the buffer size is 5*10^5^.

## 4. Experiments

### 4.1. War game platform

To verify the effectiveness and generalizability of our hierarchical RNN-based graph MADDPG method, we use the multiagent particle environment simulator All Domain Simulation (ACS) developed by China Aerospace System Simulation Technology Co., Ltd., which supports land, sea, and air scenarios, to simulate the war game.

The ACS system is developed based on C++ standard library and Qt, and the three-bit geographic information map is developed by qgis and osg earth. There are certain hardware requirements for the running environment of the computer. In addition to CPU memory and other requirements, the loading of 3D geographic information requires certain video memory space, and a GPU with at least 4GB RAM is also required. This means that we need a relatively powerful computer to run the ACS. One of the systems used in this article has the following hardware and software specifications. CPU is on the Intel Core I7-8700K, RAM size is 32 GB, GPU is NVidia GTX 1070TI, and SDK includes Visual Studio 2013, QT5.9, OSG, Unity3D, and OSG.

The reward function is a crucial component in reinforcement learning, and it usually consists of two parts. As shown in Equation (10), the first part is related to the agent's navigation goal, where the agent receives a higher reward value as it approaches the target position. This incentivizes the agent to reach the goal faster and with higher accuracy. The second part involves the avoidance of collisions between agents. In this case, a negative reward value is assigned to the agents when they collide, which encourages them to adopt safe and collision-free behaviors. Overall, the design of the reward function plays a vital role in shaping the behavior of agents and achieving the desired objectives in reinforcement learning.


(10) $$\begin{array}{l} r(t)=\{\begin{array}{ll} -\beta , & \text{ if any collisions }\\ -\sum_{i}^{N}E_{c}\left({\text{p}}_{it},{\text{g}}_{i}\right) & \text{ otherwise } \end{array} \end{array}$$

**Figure 3:**
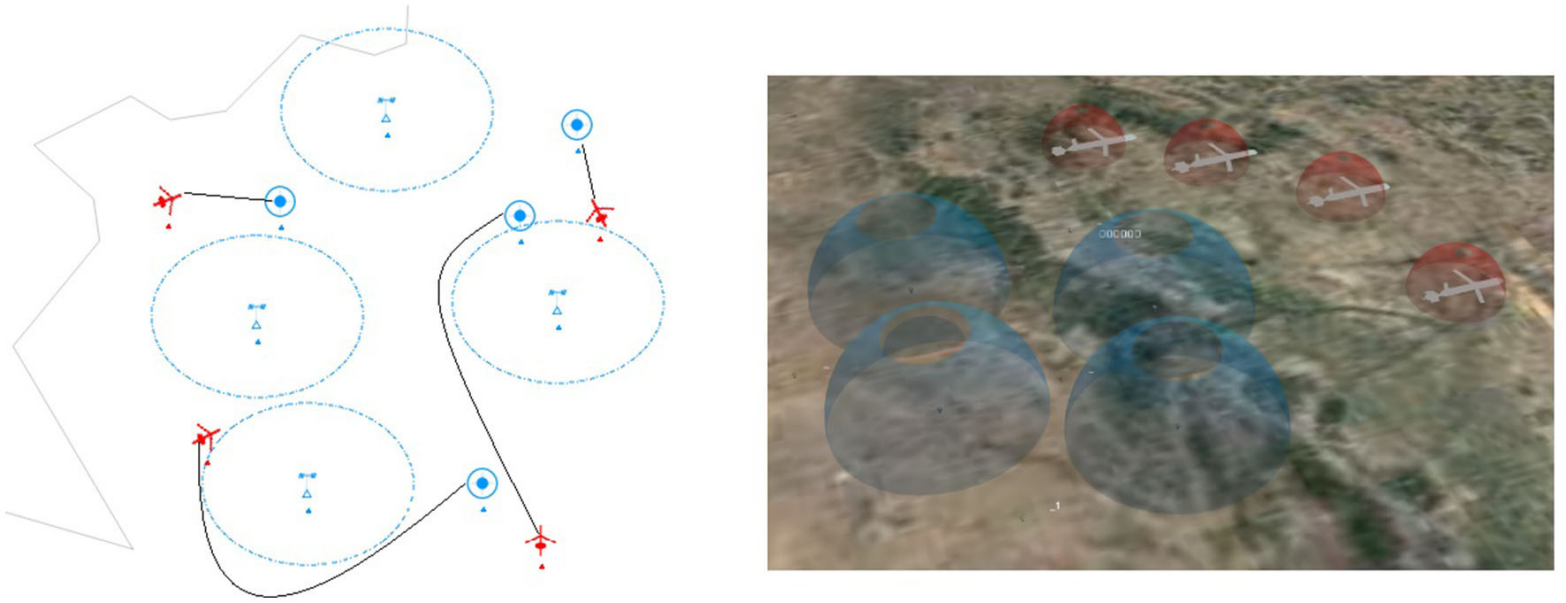
Illustrations of cooperative navigation in combat tasks. There are four radar stations, four UAVs, and four targets. The UAVs need to reach the destination without being cooperatively detected by the radar detectors.

As shown in Figure 3, in this study, we mainly consider the ACS cooperative environment, compare the reward values for different approaches and compare different hierarchies' effect results. This study conducts experiments on MADDPG, RMADDPG, hierarchical RNN-based graph transformer MADDPG, and graph MADDPG with transformers of different levels in four different test environments. A total of 25,000 rounds of training and 5,000 rounds of testing are used, and each round has 25 steps in length.

This study designs the MADDPG algorithm model combined with a graph and transformer and uses a deterministic action policy, namely, *a* = πθ(*s*). The network structure is divided into two parts: actor and critic. According to the distance between the two agents, it is judged whether there is a graph connection between the agents. Therefore, as shown in Figure 2, the input of the neural network is 3 × 2. After two layers of the graph convolutional network, it passes through two subfully connected layers, and the output is obtained. The mini-batch size during training is 1,024, the maximum round is 25,000, the update rate of the auxiliary network is 0.01, the learning rate of the value network is 0.01, and the learning rate of the policy network is 0.001. Both networks use the Adam optimizer for learning. The size of the experience pool is 1 × 10^6^. Once the data in the experience pool exceed the maximum value, the original experience data will be lost.

**Figure 4:**
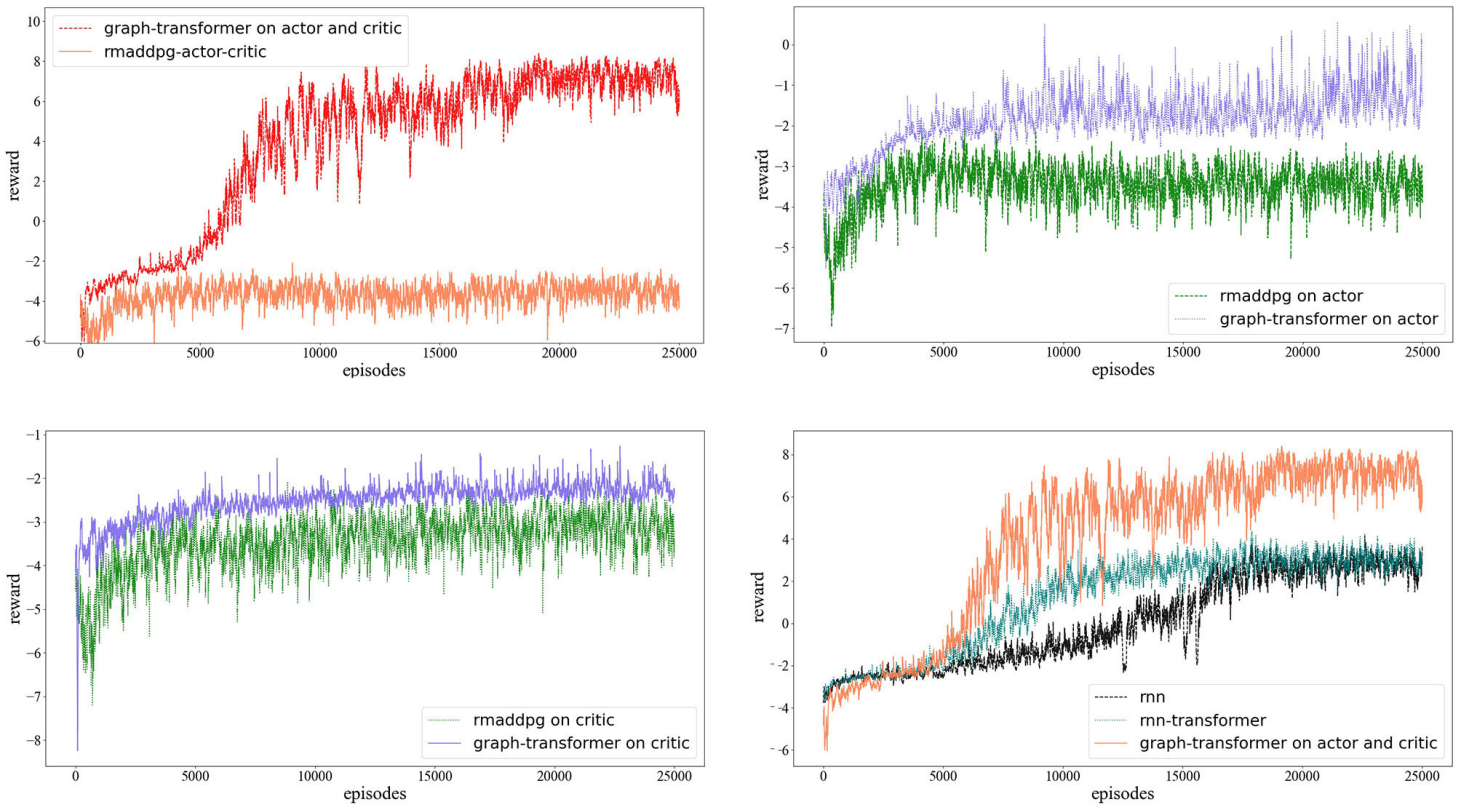
The comparison of the MARDPG, RMADDPG, graph MADDPG, and graph MADDPG with a transformer. The Y axis is the average reward of all agents for a simple navigation task. On the left, we compared the RNN, graph MADDPG, and graph MADDPG with a transformer on the actor and critic; on the right, we compared the graph MADDPG with a transformer on the actor, graph MADDPG with a transformer on the critic, and graph MADDPG with a transformer on the actor and critic.

Through the establishment of the RNN, graph-only, graph transformer on the actor and critic, graph transformer on the actor, and graph transformer on the critic, several MADDPG model structures for training are obtained. The final reward function is shown in Figure 4. The reward change graph of the three agents during each episode of training is shown. The x-coordinate represents the training number of episodes, and the y-coordinate represents the cumulative rewards of the three agents during each round of training. As the number of training sessions increases, the absolute value of the reward decreases, but the reward gradually increases. Due to the random noise in the training process, there is oscillation at any moment during the training. As shown in Figure 4, the number of training rounds reaches 10,000 rounds, the reward curves of several algorithms tend to be flat, and the overall trend is converging. Figure 4 and Table 1 show that in the experimental environment of cooperative navigation, the average reward value of the transformer change for the actor and critic networks at the same time is compared with that of RNN and transformer transformation for the actor or critic alone. The effect is better. The result of the comparison is that the effect of RNN is better than the case of only using the graph convolution operation.

**Table 1:** The proposed deep reinforcement learning framework demonstrates various indicators for each agent in cooperative navigation.

	**Average reward of cooperative navigation**			**Average number of steps needed to reach the goal**			**Time (ms)**
	**Agent1**	**Agent2**	**Agent3**	**3 agents**	**6 agents**	**9 agents**	
MADDPG	−0.775036	−0.7598686	−0.77283	400±	1,000+	1,000+	0.225
RMADDPG	3.318712	3.316456	3.318392	300±	600±	900±	2.236
Graph	−2.18555	−2.186014	−2.18629	500±	1,000+	1,000+	14.082
Graph transformer on critic	−2.271803	−2.270067	−2.27163	170±	300±	650±	13.794
Graph transformer on actor	−1.543637	−1.544357	−1.54212	120±	250±	500\pm	14.463
Graph transformer on critic and actor	**6.628632**	**6.62872**	**6.630488**	**15±**	**25±**	**55±**	14.557

The table shows that the algorithm with the highest average reward value is represented by black bold lines. Notably, the framework performs best when the graph transformer is applied to both the critic and actor.

We also found that the effect of transformer transformation on the actor and critic alone tends to be the same. This also effectively shows that the graph convolutional network has an effect on the multiagent environment, but the neural network is difficult to concentrate, the convergence speed is slow, and the effect is average, and it is slightly inferior to the RNN. In a multiagent environment, the RNN has more difficulty in expressing the relationship between multiple agents. To verify that the graph convolutional network can indeed express the relationship characteristics between agents, we added a transformer on the basis of the graph convolutional network. The results also show that the graph MADDPG with a transformer on the critic and actor algorithm has stronger stability and faster convergence.

**Figure 5:**
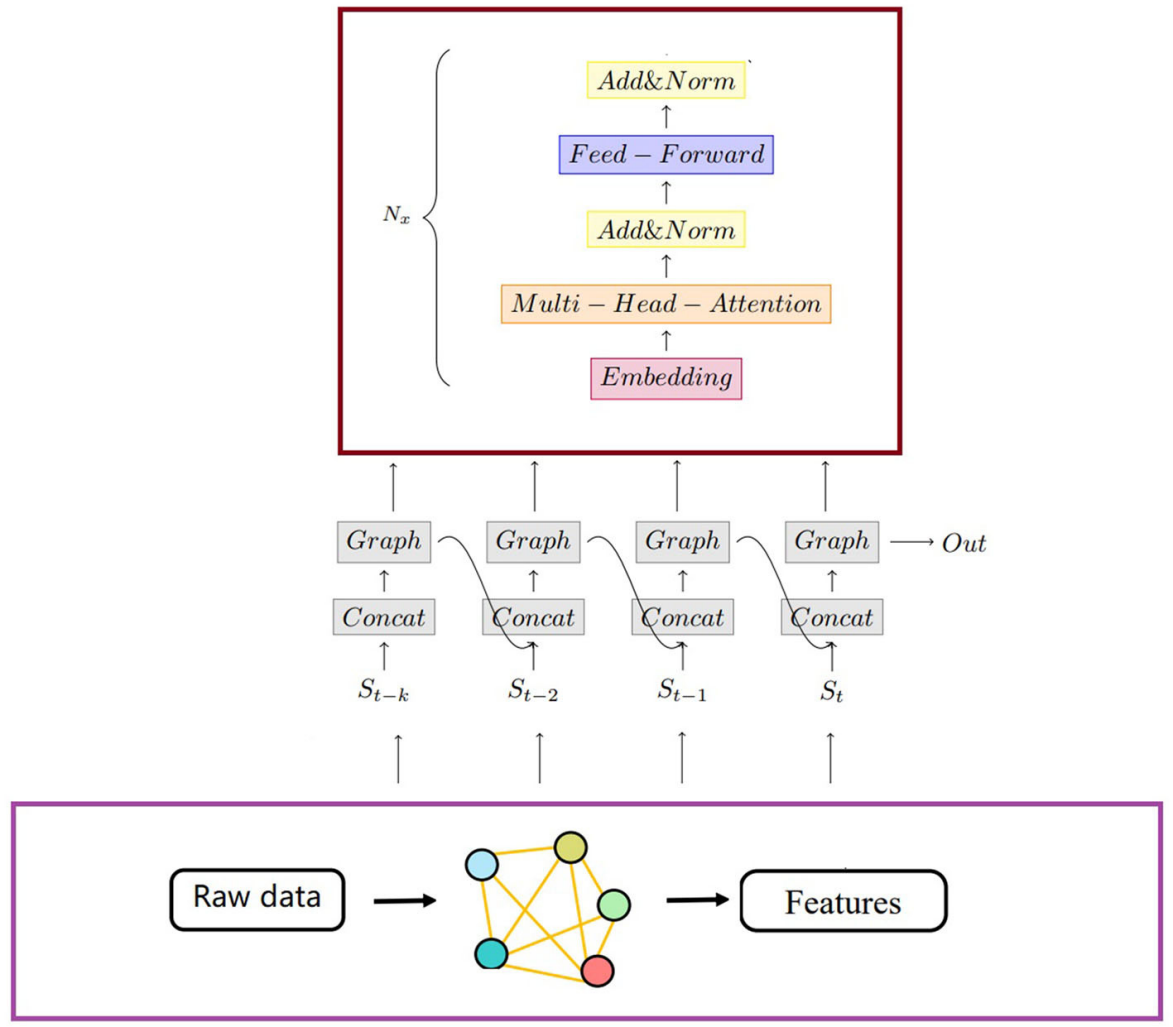
The comparison of the graph MARDPG with a transformer, RMADDPG, and RMADDPG with a transformer. The Y axis is the average reward of all agents for a simple navigation task.

Our previous results (Wei et al., 2021) proposed a hierarchical transformer MADDPG based on the RNN method, which inherited the idea of the RNN and added transformer at the same time. To verify that the graph convolution with a transformer can indeed express more features of the agent, we compared the reward values of the three sets in the experiments. They are RNN-based MADDPG, transformer-based R-MADDPG, and graph-based transformer-MADDPG. As shown in Figure 5, with the addition of a transformer, the graph-based method we proposed is significantly better than the RNN method.

**Figure 6:**
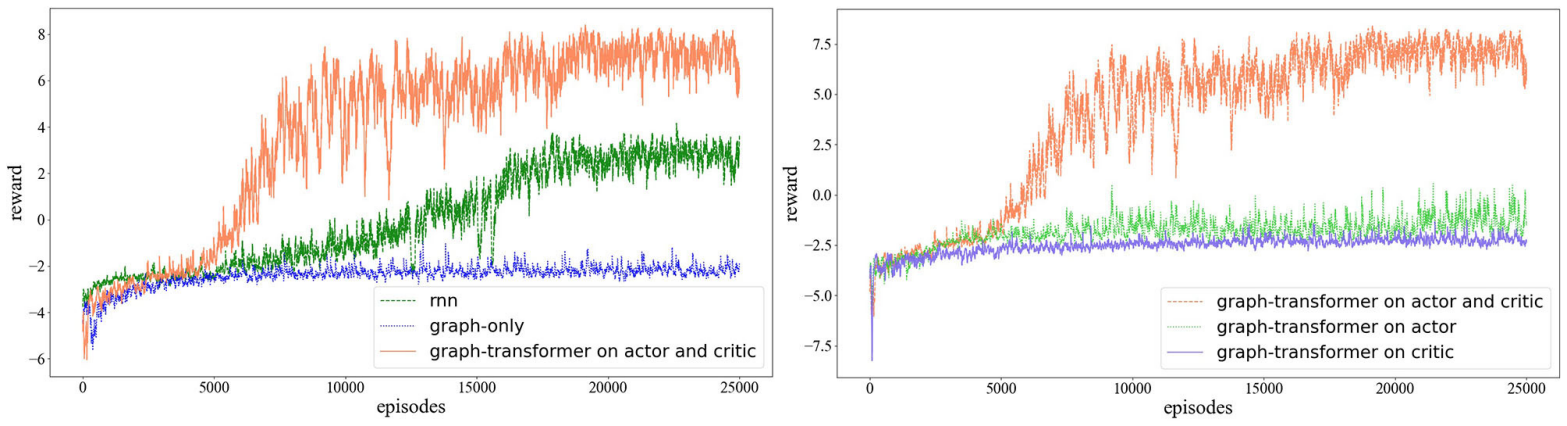
Testing in collaborative navigation environment, the **left** figure presents the comparison among rnn, graph-only and graph-transformer on actor and critic. The **right** one presents the comparison among graph-transformer on actor and critic, graph-transformer on actor and graph-transformer on critic.

As shown in Figure 6, we compare the results with R-MADDPG on the critic and actor. Based on the idea of R-MADDPG, we take RNNs as the first input layer; after the RNNs, the graph convolution layer follows. It can be seen from the results that with the training process, the agent can find the optimal value efficiently and quickly.

Through the establishment of the RNN, graph-only, graph transformer on the actor and critic, graph transformer on the actor, and graph transformer on the critic, several MADDPG model structures for training are obtained. The final reward function is shown in Figure 4. The reward change graph of the three agents during each episode of training is shown. The abscissa represents the training number of episodes, and the ordinate represents the cumulative rewards of the three agents during each round of training. As the number of training sessions increases, the absolute value of the reward decreases, but the reward gradually increases. Due to the random noise in the training process, there is oscillation at any moment during the training. From Figure 4, it can still be seen that after the number of training rounds reaches 10,000 rounds, the reward curves of several algorithms tend to be flat, but in the case of the graph transformer on the actor and critic, the average reward value increases faster. The average reward value of performing transformer changes on the actor and critic networks is comparable to that of R-MADDPG, performing transformer transformation on the actor or critic alone. This also effectively shows that graph convolution has an effect on the multiagent environment, but the neural network is difficult to concentrate, the convergence speed is slow and the effect is average, and it is slightly inferior to the RNN. In a multiagent environment, the RNN has more difficulty expressing the relationship between multiple agents. To verify that graph convolution can indeed express the relationship characteristics between agents, we added a transformer on the basis of graph convolution. The results also show that the graph MADDPG with a transformer on the critic and actor algorithm has stronger stability and faster convergence.

### 4.2. Results analysis

Figure 4 and Table 1 illustrate the results in ACS environments. We analyze the results in detail below. In the cooperative navigation environment, for our proposed approach, the rewards are shared across neighboring steps; thus, an agent's critic does not need to focus on the information from specific steps to calculate its expected rewards. On the other hand, for rewards in the competitive environment, the agents are tied to understanding the neighboring step's observations. This explains why there is an obvious improvement and the MADDPG completely breaks down, as knowing information from another specific step is crucial in predicting expected rewards.

In the experiment, we add the time duration by the agent to reach the destination and the average number of steps cost in each episode. As shown in the table, for the method MADDPG, RMADDPG cost more steps for reaching the destination. We recorded the trajectories of each agent, as shown in the figure above, using the GPG method. When an agent gets stuck in a corner, it ceases exploration and may become more chaotic if further trapped. However, with the incorporation of graph-transformers and RNNs in our method, the agent is better equipped to navigate obstacles presented by critics and actors.

## 5. Conclusion

In this study, we focus on the problem of multiagent in a 3D situation environment for electronic warfare. Graph convolution is introduced to establish the relation function between agents. Furthermore, in order to highlight the relationship between graph nodes, attention mechanism is introduced. To give consideration to the continuity of the agent in the dimension of time, the recurrent neural network is introduced. The proposed method with RNNs and a graph transformer network are highly efficient for multiagent reinforcement learning. To verify this, the experiments showed that the graph transformer with RNNs is capable of enabling MADDPG to reduce the cost of exploration and multiagent exploration.

## Data availability statement

The raw data supporting the conclusions of this article will be made available by the authors, without undue reservation.

## Author contributions

XW: writing—original draft and methodology. WC and LY: writing—reviewing. XH: conceptualization and methodology. ZT and BW: writing—reviewing and editing. All authors contributed to the article and approved the submitted version.
